# Integrating Raman spectroscopy and optical meters for nitrogen management in broccoli seedlings

**DOI:** 10.3389/fpls.2025.1613503

**Published:** 2025-07-18

**Authors:** Lorenza Tuccio, Sonia Cacini, Giulia Arati, Carmelo Distefano, Silvia Traversari, Giacomo Fontanelli, Gina Rosalinda De Nicola, Paolo Matteini

**Affiliations:** ^1^ ”Nello Carrara” Institute of Applied Physics (IFAC), National Research Council (CNR), Sesto Fiorentino, Italy; ^2^ CREA Research Centre for Vegetable and Ornamental Crops, Council of Agricultural Research and Economics, Pescia, Italy; ^3^ Research Institute on Terrestrial Ecosystems (IRET), National Research Council (CNR), Pisa, Italy; ^4^ University of Florence, Department of Agri-Food Production and Environmental Sciences (DAGRI), Florence, Italy

**Keywords:** Raman spectroscopy, Dualex, Multiplex, nitrogen, nitrate, chlorophyll, polyphenols

## Abstract

Raman spectroscopy enables non-destructive detection of nitrates and other nitrogen-related biochemical markers, including chlorophyll and polyphenols, with unparalleled specificity and sensitivity. Integrating Raman spectroscopy with proximal optical sensors, such as Dualex (Dx) and Multiplex (Mx), offers a transformative approach to precision nitrogen management in broccoli seedlings, complementing their ability to rapidly estimate nitrogen balance indices and key vegetation compounds. The integration demonstrated strong correlations between Raman spectral bands, optical indices, and biochemical parameters across varying nitrogen levels, enhancing the precision of nitrogen status assessment, resulting in a robust, scalable, and information-rich system. By combining molecular-level detail with practical field applications, this hybrid strategy represents a significant advancement in sustainable agriculture. Future research will explore the applicability of this integrated methodology to other plant species.

## 1 Introduction

The overuse of chemical fertilizers in agriculture has become a critical challenge for sustainable farming practices. Excessive nitrogen (N) fertilization not only leads to environmental issues such as nitrate leaching but also disrupts plant physiology, increasing susceptibility to pests and diseases, which in turn escalates pesticide use (Dordas, 2008). Achieving a precise N management requires accurate and efficient tools for real-time monitoring of plant health and nitrogen status.

Monitoring leaf N content is fundamental for optimizing fertilization strategies and ensuring agricultural productivity (Demotes-Mainard et al., 2008; Han et al., 2021). Traditional methods, such as Kjeldahl digestion and Dumas combustion, while accurate, are destructive, time-consuming, and unsuitable for large-scale or real-time applications. In contrast, optical techniques offer non-destructive alternatives that enable rapid and repeated measurements. Proximal optical sensors, like Dualex (Dx) and Multiplex (Mx), have been widely used to estimate leaf N content by analyzing key plant compounds such as chlorophyll and flavonoids. These compounds are critical indicators of plant nutritional status and play essential roles in photosynthesis, growth, and stress response (Muñoz-Huerta et al., 2013).

The Dx device is a leaf-clip sensor with a 6-mm diameter probe that estimates chlorophyll (DxChl) content based on leaf transmittance and measures epidermal flavonoids (DxFlav) and anthocyanins (DxAnth), using the chlorophyll fluorescence screening method (Cerovic et al., 2012). Additionally, the Nitrogen Balance Index (NBI), calculated as the ratio of DxChl to DxFlav indices, has proposed as a reliable estimate of leaf N content (Dong et al., 2021; Tuccio et al., 2022). The Mx device, on the other hand, collects information from a larger area, including entire leaves, plants, or even crops, and utilizes chlorophyll fluorescence to measure indices such as MxChl, MxFlav, and MxAnth (Diago et al., 2016).

Despite the effectiveness of these traditional optical sensors, their reliance on indirect correlations and additional parameters, such as leaf mass per area (LMA) (Bracke et al., 2019a; Tuccio et al., 2022), can limit their precision and real-time applicability. Recent advancements in optical sensing, particularly Raman spectroscopy, offer new opportunities to overcome these limitations. Raman spectroscopy provides a detailed molecular fingerprint of plant compounds by measuring the vibrational energy shifts of molecules (Saletnik et al., 2022; Park et al., 2023), providing in turn a plethora of spectral information, not limited to a few indices. This capability enables, among others, the direct and non-invasive assessment of N-related biochemical markers, such as chlorophyll, carotenoids, and polyphenols, offering both specificity and sensitivity. Previous literature has demonstrated that Raman spectroscopy allows for the early detection of plant stress and nutrient imbalances (Rys et al., 2015; Altangerel et al., 2017; Gupta et al., 2020; Sanchez et al., 2020), making it a valuable tool for precision agriculture.

Integrating an emerging technology in the agrifood area, such as Raman spectroscopy, with well-established optical sensors like Dx and Mx can represent a promising step forward in agricultural phenotyping. By combining the rapid and non-invasive nature of traditional sensors with the high-content diagnostic power of Raman spectroscopy, it becomes possible to monitor N status and overall plant health with greater accuracy and efficiency. This integration addresses the growing need for scalable, precise, and sustainable solutions in modern agriculture, especially as the sector faces increasing demands for productivity amid environmental constraints.

This study investigates the potential of combining Raman spectroscopy with Dx and Mx sensors to estimate leaf N content and monitor key biochemical compounds in broccoli seedlings cultivated in an experimental greenhouse. By leveraging the strengths of these complementary technologies, this research aims to enhance N management practices, improve plant health monitoring, and contribute to the broader goals of agricultural productivity and sustainability.

## 2 Materials and methods

### 2.1 Greenhouse experiment

**Figure 1:**
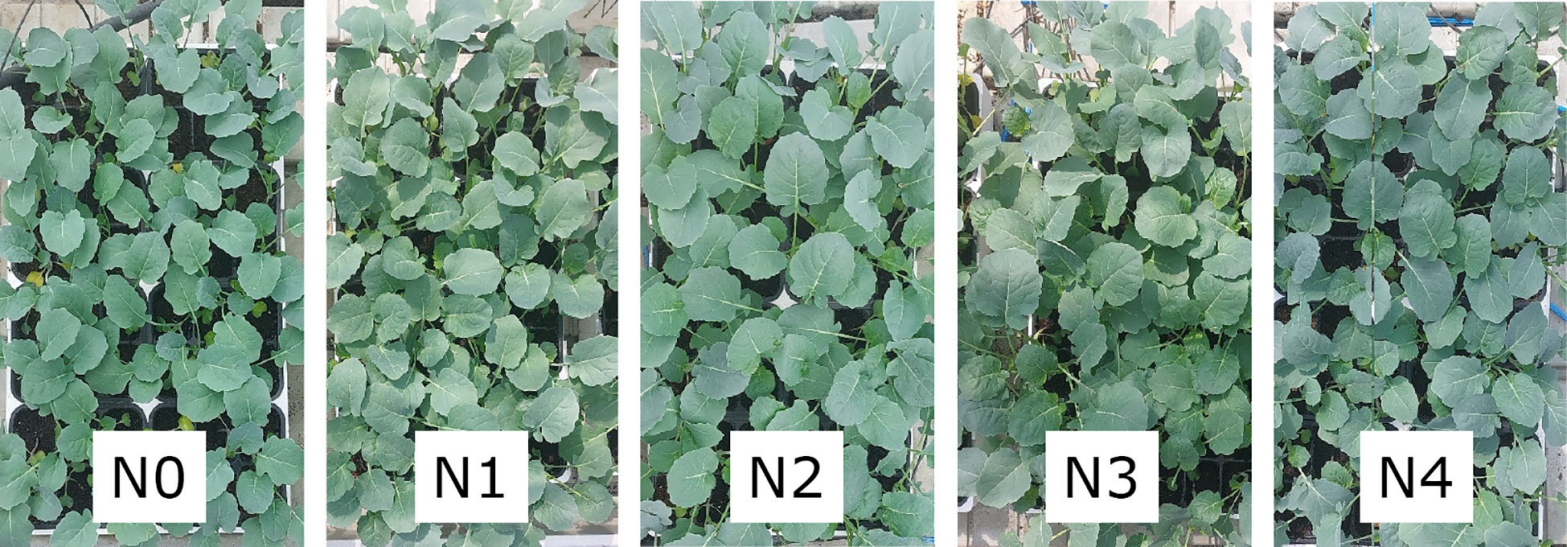
Broccoli seedlings under different N fertilization treatments: 0 mM (N0), 4.5 mM (N1), 9.0 mM (N2), 15.0 mM (N3), and 22.5 mM (N4) N.

The experiment was conducted in a greenhouse of the Research Centre for Vegetable and Ornamental Crops, Council of Agricultural Research and Economics, in Pescia, Italy, during the spring season 2024. The substrate was a mixture of 60/40 v/v dark peat/blond peat (TecnoGrowth^®^ Professional, Tercomposti S.p.A., Calvisano, BS, Italy) with an average N concentration of 12.4 g kg^-1^, determined by a FlashSmart™ NC Soil elemental analyzer (Thermo Fisher Scientific, Waltham, MA, USA). Broccoli (*Brassica oleracea* L. var. *italica* cv. Naxos F1) seedlings were planted in seed trays the 10^th^ of April 2024 and then grown in a greenhouse under different N fertilization treatments at 0, 4.5, 9.0, 15.0 and 22.5 mM N (N0, N1, N2, N3 -standard dosage according to local nursery grower practices, N4 respectively) maintaining a constant N-NO_3_/N-NH_4_ ratio of 2.75 and using a standard nutritive solution for leafy vegetables fertigation regarding to phosphorous, potassium and other micro and meso-elements (**Figure 1**). No other treatment was provided to the analyzed plants. Each treatment consisted of 4 replicates for a total of 84 seedlings per treatment. The measurement session occurred 35 d after the sowing date (DAS). Leaf collection occurred within the 10:00 – 11:00 am time interval. Water was supplied through fertigation as a function of microclimate conditions (roughly at least once a day), maintaining constant substrate moisture. Microclimate conditions were monitored every 5 minutes by a portable weather station (Zentra ZL6 datalogger, Meter Group., Pullman, WA, USA) equipped with an air temperature (T) and relative humidity sensors (RH) (VP-3; Decagon Em50; Decagon Devices Inc., Pullman, WA, USA) and a pyranometer for solar radiation measurement (Pyr sensor, Meter Group., Pullman, WA, USA). Temperature was in the range of 5.8 – 41.6°C (medium T in the period, 22°C), medium RH amounting to 60% and light intensity amounting as medium value to 246 W m^-2^ (maximum value recorded 1,007.4 W m^-2^ close to the end date of the experiment).

### 2.2 The Dualex and Multiplex optical sensors

Each leaf was analyzed with the Dualex Scientific+ (Force-A, Orsay, France) and the Multiplex (Force-A, Orsay, France) optical sensors. The leaves were measured under ambient lighting at 25°C and within 15 min of sampling. Dx chlorophyll content (DxChl) is determined by the ratio between the transmittance at 850 nm (reference signal that is not absorbed by chlorophyll) and at 710 nm. Epidermal flavonoids (Flav) are calculated by the logarithm of the ratio between chlorophyll fluorescence excited at 650 nm and 375 nm (which is attenuated by the epidermal compounds). The ratio of DxChl over DxFlav represents the nitrogen balance index (NBI), which has been shown to account for N changes in the leaf. In our experiment, two Dx readings per leaf from the upper leaf blade and lateral to the midrib of both adaxial and abaxial sides of the leaf were carried out and averaged, and the relevant DxChl, DxFlav, and DxNBI values were used for further consideration. Multiplex chlorophyll content (MxChl) is calculated from the SFR_G_ index accounting from the ratio FRF_G_/RF_G_ (between far-red and red fluorescence signals under a green excitation), while the flavonoid content (MxFlav) is calculated as the logarithm of the FRF_R_/FRF_UV_ ratio (between far-red fluorescent signals obtained under a red and a UV excitations). The MxNBI is achieved by the FRF_UV_/RF_G_ ratio (between far-red fluorescence signal under a UV excitation and the red fluorescence signal under a green excitation). A single reading from the adaxial side of a whole leaf was collected and used for further consideration. A total of 10 leaves per replica were analyzed.

### 2.3 Raman measurements

Raman spectra within the 400 ÷ 2500 cm^-1^ range were collected with a portable spectrometer (miniRaman Dual, LightNovo, Denmark) using a 785 nm laser source coupled with a laptop for parameter setting and data visualization under the proprietary software. The spectrometer was equipped with a f=30 mm lens (NA=0.05) producing a 50 mm wide spot and to which it was screwed a custom-made aluminum perforated spacer to allow collecting the maximum signal at the leaf plane surface and maintain a 90° geometry between the irradiation direction and the leaf surface. An acquisition time of 500 ms with 10 accumulations and 97.32 mW of output power was employed. The leaves, previously measured by Dx and Mx, were analyzed by Raman spectroscopy under the same conditions. Similar to the Dx measurements, a single spectrum was acquired per leaf from the upper leaf blade and lateral to the midrib of the adaxial side. A total of 10 measurements (one per leaf) per replica for a total of 40 measurements per treatment, were collected, averaged, and used for further evaluation. This approach helps to mitigate spatial variability and improves the representativeness of the spectral data. Raman spectra were preprocessed before analysis according to Ref (Matteini et al., 2025). Raman spectra of kaempferol and quercetin and their glucoside derivatives (Extrasynthese, Genay, France) were collected under a LabRAM HR Evolution spectrometer (Horiba, Lille, France) working in back-scattering geometry and equipped with an excitation laser source at 785 nm (de Angelis et al., 2025), focused through a ×10 objective from 0.1 M stock solutions in dimethyl sulphoxide.

### 2.4 Analytical determination of chlorophyll and nitrogen in leaf

After optical measurements, each leaf was weighed to determine the fresh weight (FW) and dry weight (DW), by oven drying until reaching constant weight at 70°C, and then milled for subsequent destructive analyses. Before drying, the same leaves were used for the measurement of leaf area (LA) by a leaf area meter (WinDIAS Image Analysis System, Delta-T Devices, Cambridge, UK) and for the determination of the Leaf Mass per Area (LMA), namely the DW on LA ratio. Chlorophyll a (Chl a) and b (Chl b) and total phenols concentrations were determined by spectrophotometer analysis (Evolution™ 300 UV–Vis Spectrophotometer, Thermo Fisher Scientific Inc., Waltham, MA, USA) after fresh leaf samples extraction in methanol (99%), keeping samples in the dark at -20°C for 48 h, renewing the solution after 24 h, and reading the absorbance at 665.2, 652.4, 470.0 and 320.0 nm, respectively. Chl a, Chl b, and total chlorophyll (Chl_a+b_) were then calculated as described by (Lichtenthaler and Buschmann, 2001), while total phenols (Total_Phenols) were calculated according to (Maggini et al., 2018). Total Kjeldahl Nitrogen (TKN) concentration was measured by Kjeldahl method after phospho-sulphuric acid digestion (Massa et al., 2016). Nitrate nitrogen (N-NO_3_) leaf concentration was determined by the spectrophotometric assay using the salicylic-sulfuric acid method described by (Cataldo et al., 1975) after 1 h water extraction of dry leaf tissue.

### 2.5 Statistics

Statistical analysis was carried out with OriginPro 2024 version 10.1.0.178 software (OriginLab Corporation, Northampton, MA). Mean data values were analyzed using ANOVA and compared with the all-pairwise multiple comparison Tukey’s test. *P* values of <0.05 were considered statistically significant.

## 3 Results and discussion

### 3.1 Raman spectral features of broccoli seedling leaves

**Figure 2:**
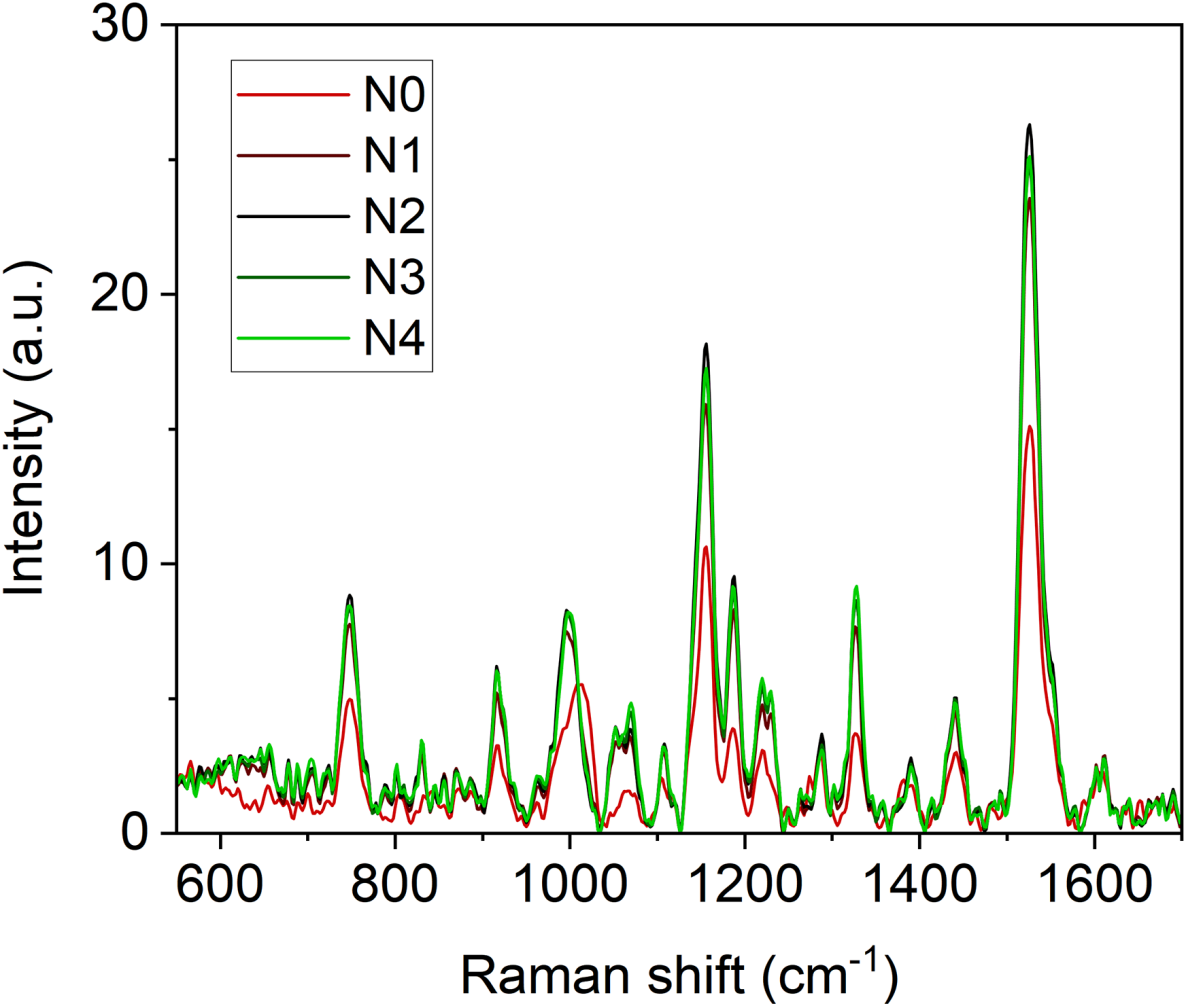
Averaged (n = 4) Raman spectra of broccoli seedling leaves at different N fertilization doses (N0 **=** 0 mM, N1 **=** 4.5 mM, N2 **=** 9.0 mM, N3 **=** 15.0 mM, N4 **=** 22.5 mM).

The Raman spectra of broccoli seedling leaves exhibit a distinctive signature characteristic of green leafy vegetables when excited under a 785 nm light source (**Figure 2**). This signature is primarily defined by the vibrational modes of carotenoids, chlorophyll, and polyphenols. Carotenoids contribute bands at 1003, 1156, 1188, 1218 and 1526 cm^-1^ (Bhosale et al., 2004; Baranski et al., 2005; Ibarrondo et al., 2016; Park et al., 2023). Chlorophyll (a and b) is represented by major bands at 747, 917, 992, 1188, 1220, and 1328 cm^-1^, with additional shoulders at 1141 and 1555 cm^-1^ (Kagan and Mccreery, 1995; Ishihara and Takahashi, 2023). Polyphenols display bands mainly in the ranges of 550 – 650, 1245 – 1321, 1370 – 1420, 1586–1670 cm^-1^ (Jurasekova et al., 2006, Jurasekova et al., 2014; Gamsjaeger et al., 2011; Pompeu et al., 2018; Bock et al., 2021).

### 3.2 Correlation between Raman signals, optical meters, and biochemical parameters

**Figure 3:**
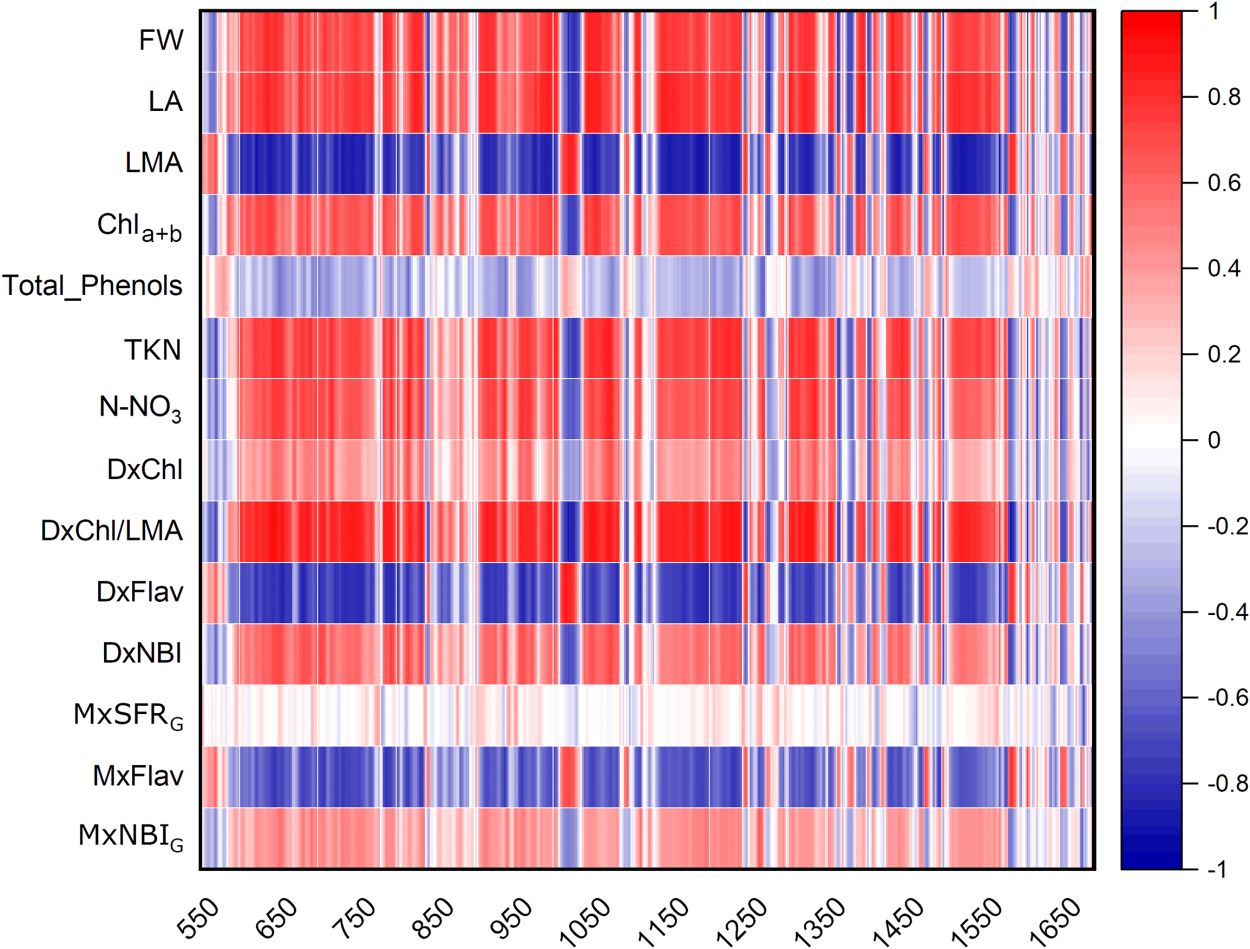
Heatmap of Pearson**’**s correlation coefficients r between Raman bands of broccoli under N0-N4 fertilization dosages and different parameters: biometric features (FW, fresh weight; LA, leaf area; LMA, leaf mass per area); extracted compound levels (Chl_a+b_, Total_Phenols, TKN, N-NO_3_); Dx and Mx indexes (DxChl, DxChl/LMA, DXFlav, DxNBI, MxSFR_G_, MxFlav, MxNBI_G_). A positive correlation (r **>**0) is represented by red tones, while a negative correlation (r **<**0) is depicted in blue tones.

**Figure 4:**
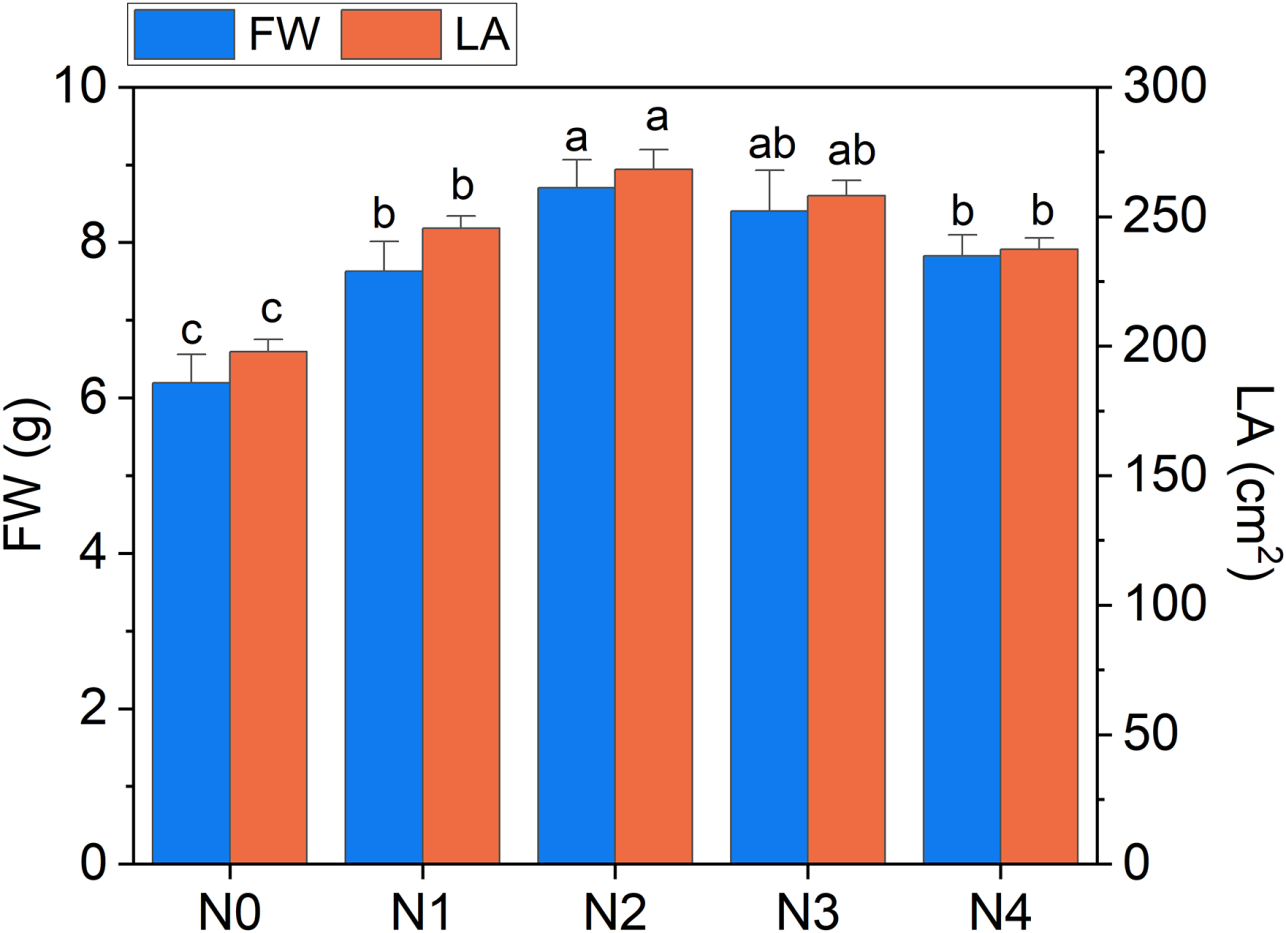
Fresh weight of broccoli seedling leaves (FW) and leaf area (LA) as a function of N dosages. Statistical analysis was performed through one-way ANOVA. Bars represent the means (n = 4) + SEs. Different letters indicate a statistically significant difference according to *post-hoc* Tukey HSD method (p<0.05).

**Figure 5:**
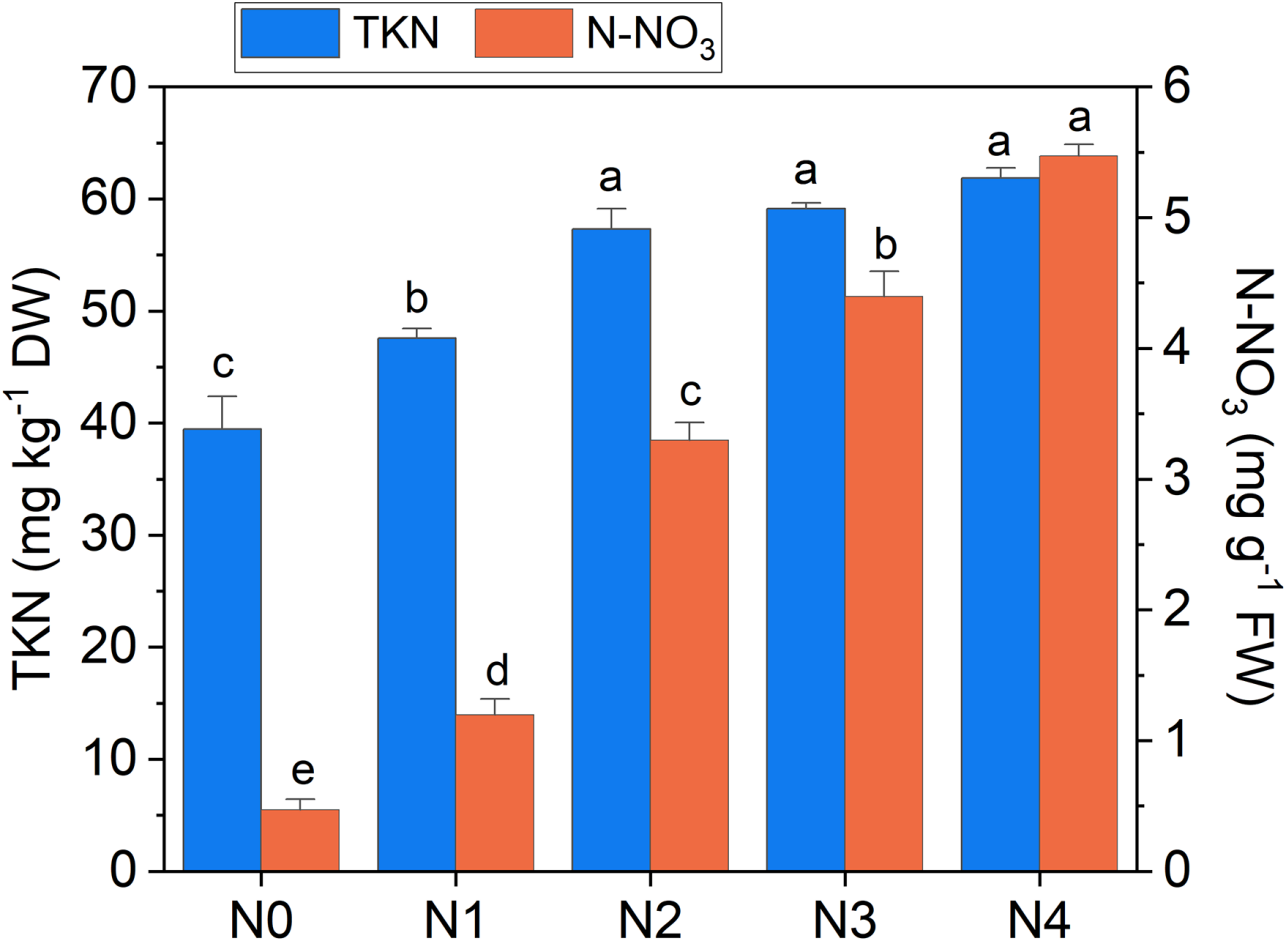
Total Kjeldahl nitrogen (TKN) and nitrate nitrogen (N-NO_3_) concentration in broccoli seedling leaves as a function of N doses. Statistical analysis was performed through one-way ANOVA. Bars represent the means (n = 4) + SEs. Different letters indicate a statistically significant difference according to *post-hoc* Tukey HSD method (p<0.05).

**Figure 6:**
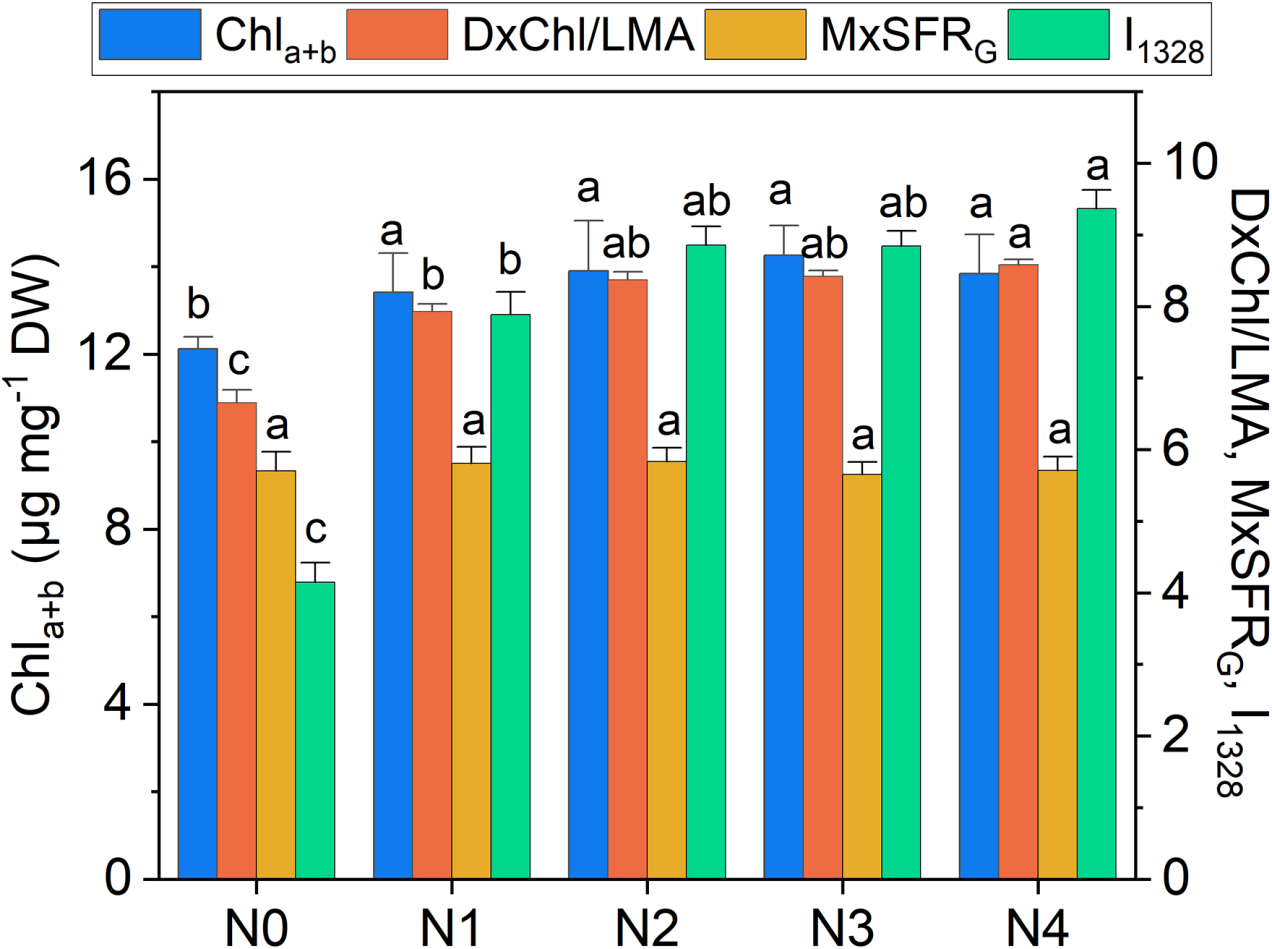
Extracted total chlorophyll content (Chl_a+b_), DxChl/LMA, MxSFR_G_ indexes, and Raman intensity of the 1328 cm^-1^ band (I_1328_) as a function of N doses. Statistical analysis was performed through one-way ANOVA. Bars represent the means (n = 4) + SEs. Different letters indicate a statistically significant difference according to the *post-hoc* Tukey HSD method (p<0.05).

The heatmap in **Figure 3** illustrates the relationships between Raman spectral frequencies and various parameters, including biometric metrics (FW, LA, and LMA), concentration of plant compounds (Chl_a+b_, Total_Phenols, TKN, and N-NO_3_) obtained through destructive analyses, and indices derived from Dx and Mx measurements (DxChl, DxChl/LMA, DxFlav, DxNBI, MxSFR_G_, MxFlav, MxNBI_G_) on the same samples. A notable observation is the close correlation pattern between FW and LA on one side, and the concentrations of extracted chlorophyll (Chl_a+b_), TKN, and N-NO_3_ on the other. Higher N levels are often positively correlated with increased total chlorophyll content, as N is a fundamental component of chlorophyll molecules and essential for photosynthesis (Wen et al., 2019). This increase in chlorophyll typically enhances photosynthetic efficiency, leading to greater leaf expansion (larger LA) and higher biomass accumulation (FW). Optimal N levels generally promote balanced growth, while insufficient or excessive nitrogen can negatively impact chlorophyll synthesis, leaf area, and fresh weight (Boussadia et al., 2010). Accordingly, we observe an increase in LA and FW with higher fertilization levels up to N2, followed by a slight decrease due to excessive N supply (**Figure 4**). While N-NO_3_ levels continue to rise from N0 to N4, TKN reaches a saturation stage starting at N2 (**Figure 5**), negatively affecting biomass performance (**Figure 4**), and chlorophyll content (Chl_a+b_ plateaus for N > 2, **Figure 6**). LMA clearly distinguished N0 from the other doses (**Supplementary Figure S1**), showing a significantly higher value at N0 and stabilizing at a lower, statistically similar level for N1–N4. This trend aligns with the observed increases in LA and FW up to N2, as nitrogen availability promotes leaf expansion and biomass accumulation, leading to thinner leaves and a lower LMA. Beyond N2, excessive N negatively impacts biomass production without further affecting LMA.

### 3.3 Raman spectroscopy for chlorophyll and flavonoid monitoring

Chlorophyll variations across fertilization levels are well-tracked by DxChl/LMA as well as by the 1328 cm^-^¹ Raman chlorophyll band (I_1328_, **Figure 6**). Notably, both DxChl/LMA and I_1328_ show improved differentiation among N levels, surpassing even the spectrophotometric analysis. We note that the correlation between Raman and DxChl significantly improves when DxChl is normalized by LMA (R^2^ = 0.31 for DxChl vs. I_1328_; R^2^ = 0.84 for DxChl/LMA vs. I_1328_, **Supplementary Figure S2**). A similar improvement of DxChl index is observed in the correlation with total chlorophyll concentration (R^2^ = 0.10 for DxChl vs. Chl_a+b_; R^2^ = 0.60 for DxChl/LMA vs. Chl_a+b_), highlighting a dependence on leaf morphology (*e.g.*, thickness or area) with DxChl/LMA offering a clearer representation of chlorophyll concentration per unit leaf mass, as previously pointed out (Bracke et al., 2019a; Tuccio et al., 2022). It is noteworthy that other Raman bands associated with chlorophyll exhibit similar predictive behavior, albeit with lower R^2^ values (R^2^ < 0.80, **Supplementary Figure S2**). Further taking into consideration medium-high covariance between I_1328_ and extracted Chl_a+b_ (R^2^ = 0.64) and the almost absence of other competing bands in that Raman region, the 1328 cm^-^¹ frequency, which is ubiquitarian in the Raman spectrum of chlorophyll (Koyama et al., 1986; Sato et al., 1995), is here suggested as a valuable indicator for predicting chlorophyll concentration in green leaves using Raman spectroscopy, while also offering a non-destructive alternative to LMA-based assessments.

**Figure 7:**
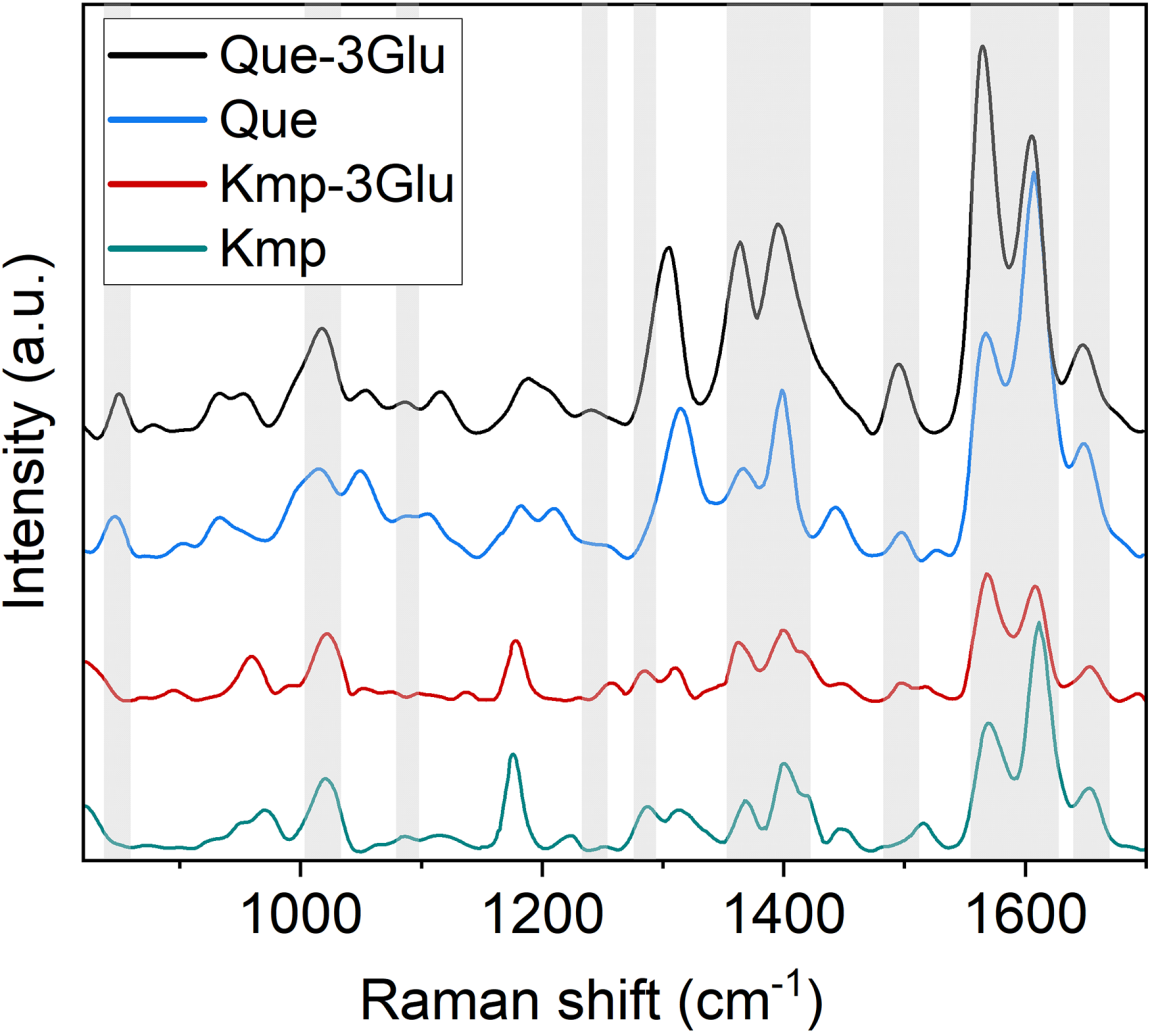
Raman spectra of kaempferol (Kmp), kaempferol-3glucoside (Kmp-3Glu), quercetin (Que), quercetin-3O glucoside (Que-3Glu). Bands involved in the correlation with DXFlav and MxFlav are highlighted by a grey box.

**Figure 8:**
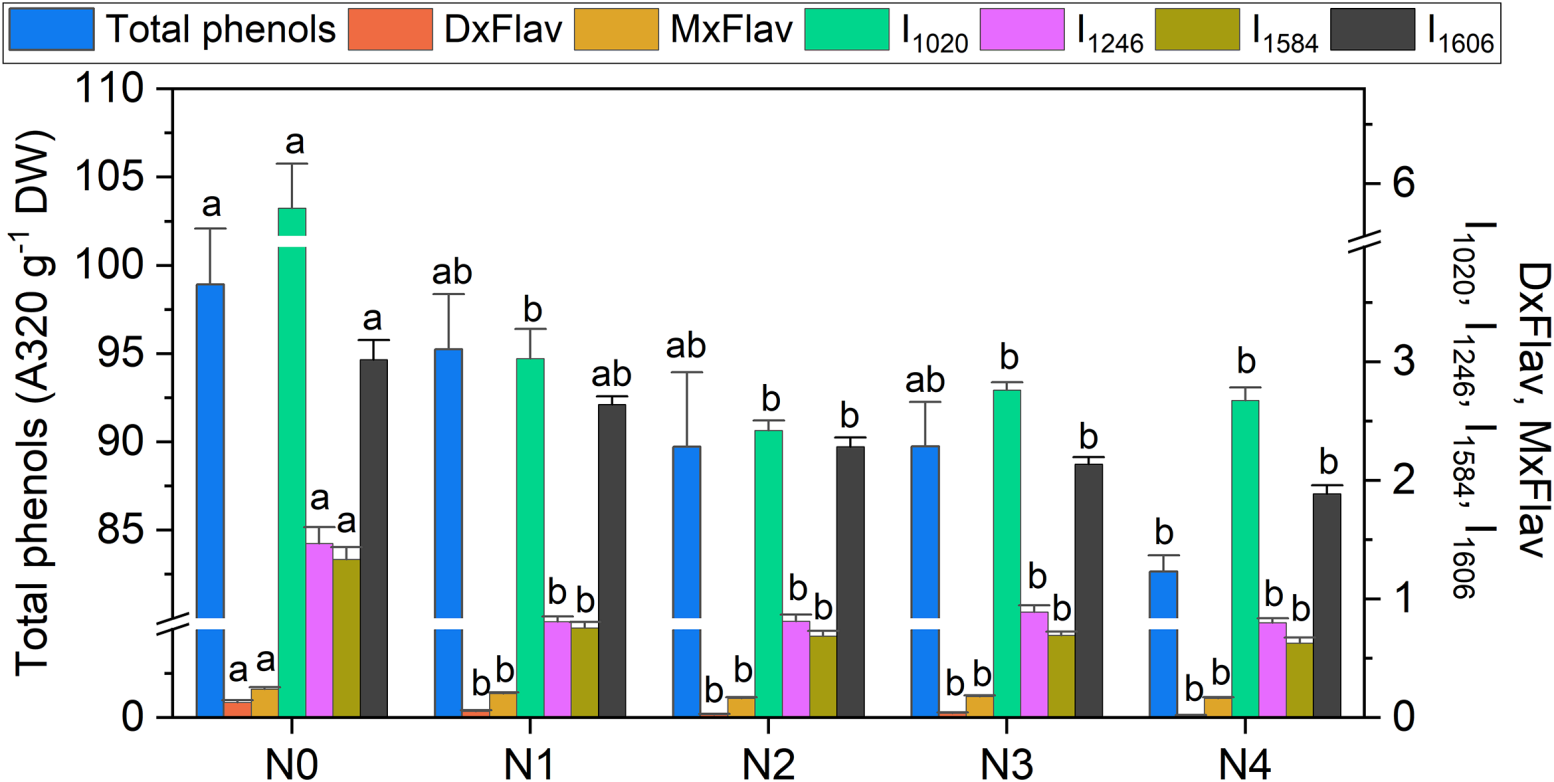
Extracted total phenol concentration, DxFlav, MxFlav indexes, and Raman intensity of 1020 cm^-1^ (I_1020_), 1246 cm^-1^ (I_1246_), 1584 cm^-1^ (I_1584_), and 1606 cm^-1^ (I_1606_) bands as a function of N dosages. A stabilization of stress-related secondary metabolism compounds above N≥N2 suggests that the N2 dose (9.0 mM) may represent a potential optimum in terms of both biomass production and metabolic balance, with a 40% reduction in N, compared to the standard dose (N3) applied by local producers. Statistical analysis was performed through one-way ANOVA. Bars represent the means (n = 4) + SEs. Different letters indicate a statistically significant difference according to the *post-hoc* Tukey HSD method (p<0.05).

High positive correlations (**Figure 3**) are observed between several Raman spectral regions and the reference Dx and Mx indices for flavonoids (DxFlav, MxFlav). Specifically, these regions are: 838–848 cm^-^¹, 1010–1030 cm^-^¹, 1092–1096 cm^-^¹, 1244–1248 cm^-^¹, 1272–1276 cm^-^¹, 1364–1422 cm^-^¹, 1474–1502 cm^-^¹, 1580–1612 cm^-^¹, and 1645–1674 cm^-^¹. A similar correlation pattern, albeit weaker, is observed with the total phenols concentration obtained through leaf destructive analysis (Total_Phenols, **Figure 3**). Dx and Mx indices are limited to detecting epidermal flavonoids, particularly those attenuating chlorophyll fluorescence due to their optical absorption in the UV-A region (specifically at 375 nm, as implemented in Dx and Mx optical sensors) (Diago et al., 2016). Broccoli are rich in flavonols such as quercetin and kaempferol and their 3O-glycoside derivatives (Vallejo et al., 2004; Cartea et al., 2011). The Raman spectrum of quercetin, kaempferol, and their 3O-glucosilates are shown in **Figure 7**, along with evidenced bands involved in the correlation with Dx and Mx. Interestingly, some main bands, such as those at ~1310 cm^-^¹, lack a positive correlation with Dx and Mx indices (**Figure 3**). This can be explained by their proximity with strong chlorophyll and carotenoid bands (**Figure 2**), which diminish or reverse their correlation with DxFlav and MxFlav in **Figure 3**. Conversely, the spectrophotometric measurement (*i.e.*, Total_Phenols) represents a broad estimate of the overall phenolic content. This explicates its additional positive covariance with certain Raman frequencies (*i.e.*, at 950 and 1684 cm^-1^ associated with phenolic compounds beyond epidermal flavonoids, such as epicatechin, gallic acid, and p-coumaric acid, which were also found in mature broccoli (Rahman et al., 2022)). These compounds typically exhibit an optical absorption below 370 nm (Csepregi et al., 2013), making them undetectable by Dx and Mx sensors. A decrease in phenolic content (Total_Phenols) is observed with increasing N fertilization dosages (**Figure 8**), which also reflects the DxFlav and MxFlav trend as well as that of selected Raman bands, such as the 1020, 1246, and 1584 cm^-^¹. Notably, DxFlav, MxFlav, and the intensities of these Raman bands align closely in differentiating among N doses, whereas variations in the 1606 cm^-^¹ intensity more closely mirror those of total phenols. This distinction arises because the first three frequencies correspond to Raman bands of quercetin and kaempferol and their glucoside derivatives (**Figure 7**), explaining their predictive value for the flavonoid content (mainly flavonols) with a strong correlation with DxFlav (R^2^ 0.65 ⨫ 0.80), and moderately high correlation with MxFlav (R^2^ 0.53 ⨫ 0.65). In contrast, the 1606 cm^-^¹ band (R^2^ = 0.52 vs. DxFlav and MxFlav) lies in a spectral region less specific to flavonoids but includes Raman features of the main part of polyphenols (Pompeu et al., 2018; Espina et al., 2022; Krysa et al., 2022). Consequently, this band (ascribed to the aromatic C=C stretching) is more ubiquitous across various polyphenol species and, therefore, appears well-suited for deriving information about the total phenolic content of the sample, as suggested elsewhere (Pompeu et al., 2018).

### 3.4 Raman-based nitrogen estimation

The inverse relationship between flavonoid content and N availability in plants has been highlighted by several studies. Flavonoid content increases under N (nitrate and ammonium) deficiency because the plant reallocates resources from protein synthesis and growth to secondary metabolite production, enhancing defense mechanisms against environmental stress (Prinsi et al., 2020; Ma et al., 2023). This relationship has been explored not only to monitor plant responses to varying fertilization levels (Tremblay et al., 2007, Tremblay et al., 2010; Agati et al., 2016), as also evidenced in this study (**Figure 8**), but also as a potential tool to quantify N content (Cartelat et al., 2005; Bracke et al., 2019b; Tuccio et al., 2022). The correlation coefficients between DxFlav and MxFlav vs. N content (N-NO_3_ and TKN) in broccoli as listed in **Table 1** reveal a moderate potential (R^2^ ~0.50), which is here surpassed using the DxChl index or the NBI values, reaching excellent performances in the case of N-NO_3_ (R^2^ = 0.81 and 0.71 for DxChl and DxNBI, respectively). The chlorophyll content is often used as an indicator of available nitrogen levels (Padilla et al., 2018) due to the almost linear relationship between these parameters (**Figure 3**), as previously mentioned. Another chance is to take advantage of NBI reflecting the ratio between chlorophyll and flavonoids, which can sometimes improve N estimates, especially under conditions of high nitrogen levels (Shi et al., 2024).

**Table 1:** Coefficients of determination (R^2^) of Dx, Mx indexes, and selected Raman bands with total Kjeldahl nitrogen (TKN) and nitrate nitrogen (N-NO_3_) concentrations in broccoli seedling leaves.

Index	N-NO_3_	TKN
DxChl	0.81^*^	0.57
MxSFR_G	0.18	0.18
DxFlav	0.48	0.50
MxFlav	0.53	0.56
DxNBI	0.71^*^	0.61
MxNBI	0.18	0.30
I_820_	0.78^*^	0.74^*^
I_1065_	0.73^*^	0.75^*^
I_1336_	0.74^*^	0.71^*^
I_692_	0.56	0.71^*^
I_1004_	0.54	0.71^*^
I_1224_	0.55	0.71^*^
I_1446_	0.59	0.74^*^
I_938_	0.67^*^	0.52
I_1268_	0.68^*^	0.43

High correlation values exceeding R^2^ = 0.65 are marked with an asterisk.

**Figure 9:**
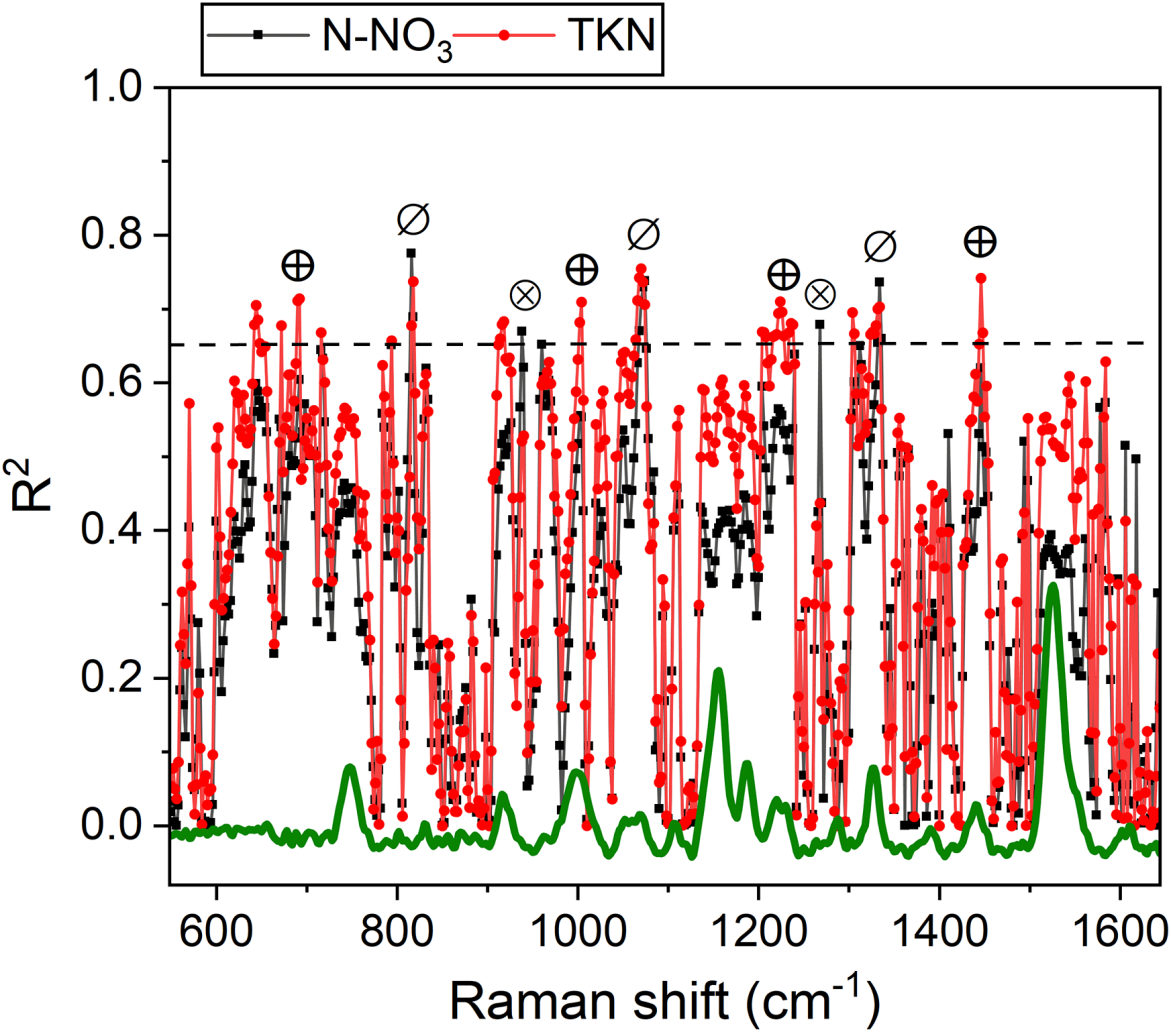
R² values for N-NO_3_ (black) and TKN (red) as a function of Raman frequencies. A dashed line indicates the R² threshold of 0.65. Symbols mark frequencies where R² > 0.65 correlations are observed with both N-NO_3_ and TKN (⊘), primarily with N-NO_3_ (⊗) or primarily with TKN (⊕).

Several Raman frequencies exhibit a correlation of R^2^ >0.65 with N-NO_3_ and/or TKN (see **Figure 9**, **Table 1**). Specifically, bands at 820, 1065 and 1336 cm^-1^ effectively correlate with both N-NO_3_ and TKN. These bands are linked to cellulose and structural cell wall components, representing the C-C and C-O stretching, the C–O–C stretching, and the C-O stretching or C-O-H bending modes of polysaccharides, respectively (Chylińska et al., 2014; Szymańska-Chargot et al., 2016; Bock et al., 2021; Payne and Kurouski, 2021). Overall, their association with N content underscores the dependence of these species on N availability, which regulates plant growth and cell wall biosynthesis (Ogden et al., 2018). The TKN demonstrates a strong correlation with bands at 692, 1004, 1224 cm^-1^ (R^2^ >0.65) and a moderate correlation (R^2^ = 0.55-0.60) with bands at 746, 916, 1160, 1530 cm^-1^ of chlorophyll and carotenoids, as well with the more generic 1446 cm^-1^ band (CH_2_ bending). This can be explained by ammonium’s direct role in metabolic pathways that enhance and regulate photosynthetic pigment synthesis and the production of CH-rich structural and storage compounds in photosystems (Chenard et al., 2005; Wen et al., 2019) (in fact TKN is limiting for chlorophyll, see **Figure 5** and **Figure 6**). Conversely, the 938 and 1268 cm^-1^ bands correlate preferentially with N-NO_3_ and may tentatively reflect C-H bending modes in aliphatic chains of lipids and fatty acids, or C-O stretching vibrations in phenolic compounds (Pompeu et al., 2018; Saletnik et al., 2022). The accumulation of these compounds can align with nitrate availability, which regulates lipid biosynthesis and phenolic metabolism (Cartea et al., 2011; Pereira et al., 2024).

**Figure 10:**
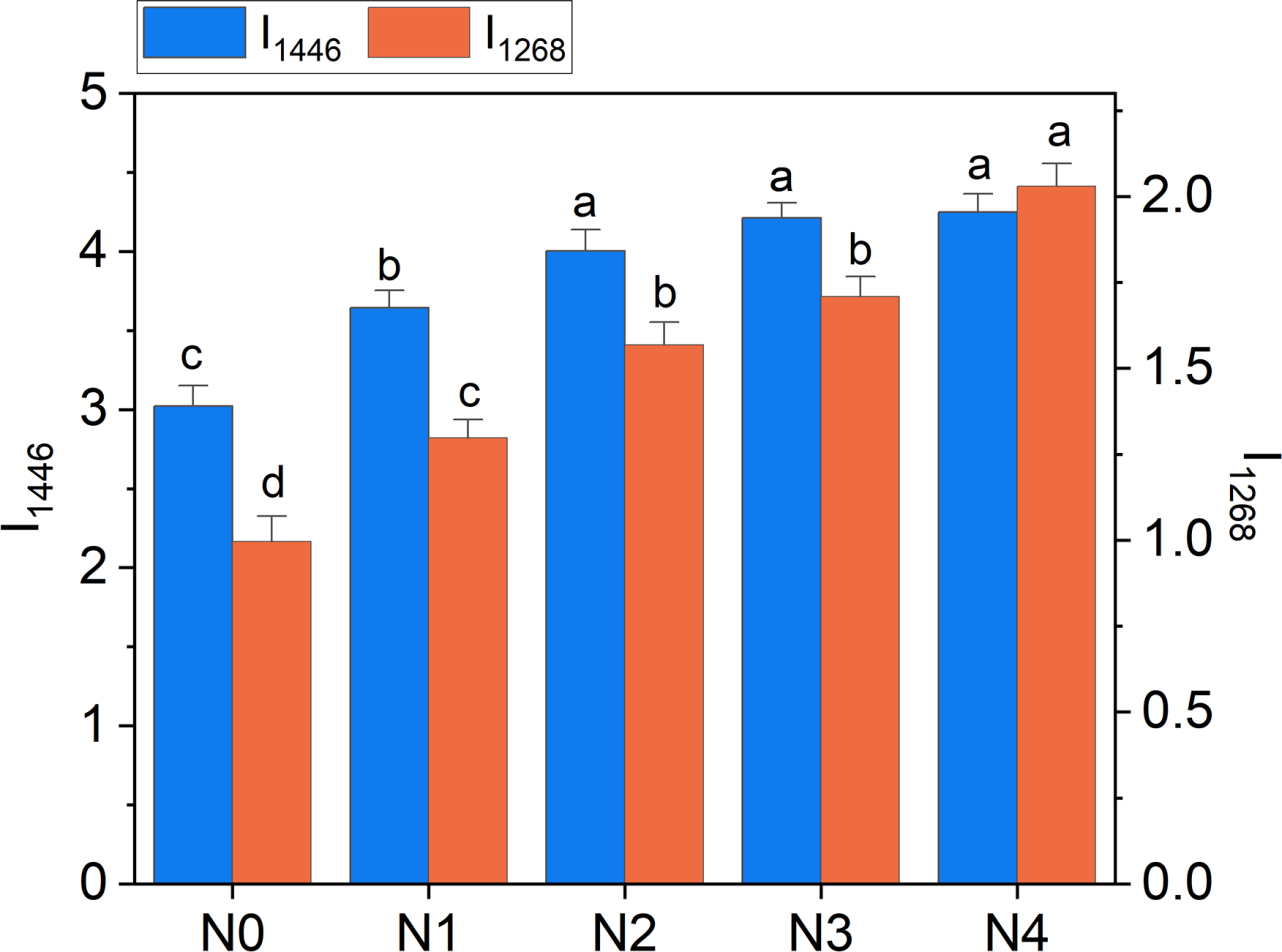
Raman intensity of 1268 cm^-1^ (I_1268_) and 1446 cm^-1^ (I_1446_) bands as a function of N doses. Statistical analysis was performed through one-way ANOVA. Bars represent the means (n = 4) + SEs. Different letters indicate a statistically significant difference according to the *post-hoc* Tukey HSD method (p<0.05).

Overall, the above considerations highlight the potential of Raman bands as effective tools for probing available nitrogen in broccoli, with performance comparable to or exceeding that of portable meters (**Table 1**). Moreover, we have evidence that a strategic selection of band intensities could provide specific insights into N-NO_3_ or TKN levels, offering valuable guidance for the optimized management of fertilizer doses. For example, the 1446 cm^-1^ band can represent an excellent reference to follow the variation in TKN, while the 1268 cm^-1^ band is willing to resemble variations in N-NO_3_ with enough accuracy, as emerges comparing **Figure 10** with **Figure 5**.

## 4 Conclusions

This study highlights the effectiveness of integrating Raman spectroscopy with standard optical meters, such as Dualex and Multiplex, to improve N management in broccoli seedlings. Raman spectroscopy provides molecular-level precision in detecting and quantifying N-related markers, including chlorophyll, flavonoids, and plant nitrogen, while integration with optical meters enables rapid, non-invasive assessments in real-time. The findings demonstrate that this hybrid strategy delivers superior accuracy compared to standalone approaches. Strong correlations were observed between Raman spectral bands and indices derived from optical meters, such as Chl, Flav, and NBI, as well as with biochemical analyses of chlorophyll and N levels. This synergy addresses individual limitations of each technology, offering a comprehensive understanding of plant nutritional dynamics. The integration provides significant practical advantages, including ease of use and scalability, making it ideal for precision agriculture. Potential influences of factors such as light interception and nutrient interaction at high nitrogen levels on spectral responses should be considered in future studies to further improve the robustness of this integrated approach. Future perspectives include validating the system across multiple broccoli cultivars and within a lower-to-intermediate N range, applying this framework to other plant species, broadening its adoption across diverse agricultural systems and contributing to sustainable farming practices.

## Data Availability

The raw data supporting the conclusions of this article will be made available by the authors, without undue reservation.
