# Supplementary material for: Integrating Raman spectroscopy and optical meters for nitrogen management in broccoli seedlings
Source: Front Plant Sci. 2025 Jul 18;16:1613503. doi: 10.3389/fpls.2025.1613503 (PMC12313702; doi:10.3389/fpls.2025.1613503)
Supplement: Supplementary file 1 [file DataSheet1.docx]

Supplementary Material


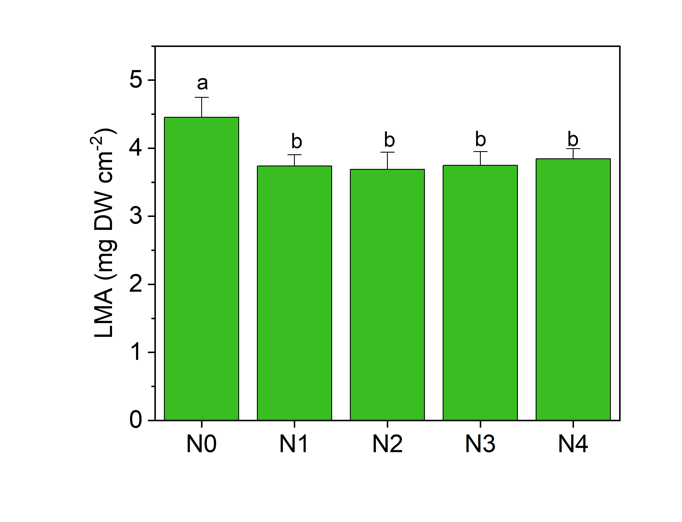


Figure S1. Leaf mass per area (LMA) as a function of N doses. Statistical analysis was performed through one-way ANOVA. Bars represent the means (n = 4) + SEs. Different letters indicate a statistically significant difference according to the post-hoc Tukey HSD method (p<0.05).


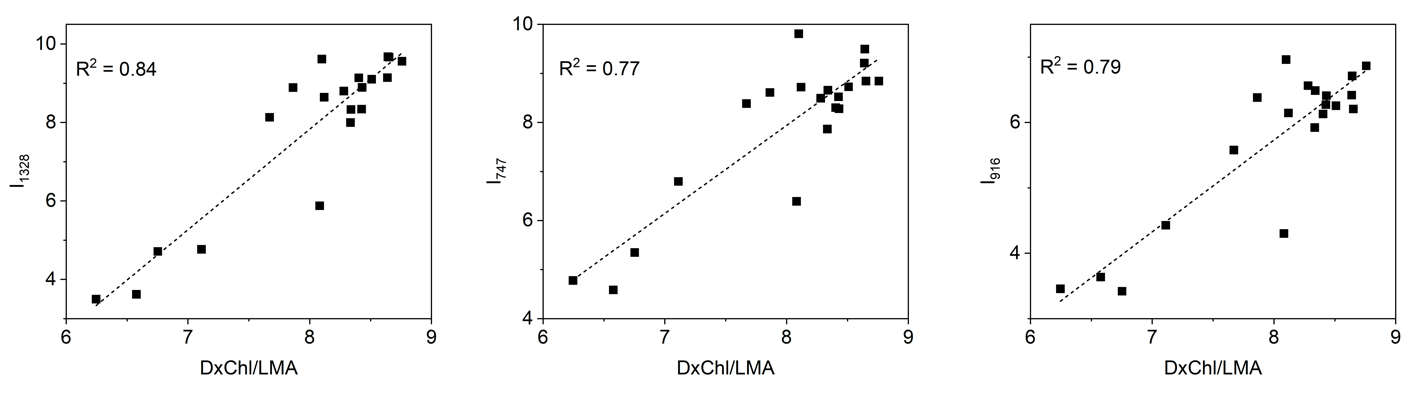


Figure S2. Relationship between DxChl/LMA and different Raman frequencies ascribed to chlorophyll.
